# The matrikine acetyl-proline-glycine-proline and clinical features of COPD: findings from SPIROMICS

**DOI:** 10.1186/s12931-019-1230-8

**Published:** 2019-11-12

**Authors:** J. Michael Wells, Dongqi Xing, Liliana Viera, Robert M. Burkes, Yixin Wu, Surya P. Bhatt, Mark T. Dransfield, David J. Couper, Wanda O’Neal, Eric A. Hoffman, Amit Gaggar, Igor Barjaktarevic, Jeffrey L. Curtis, Wassim W. Labaki, Mei Lan K. Han, Christine M. Freeman, Nirupama Putcha, Thomas Schlange, J. Edwin Blalock, for the SPIROMICS Investigators,, Neil E. Alexis, Wayne H. Anderson, R. Graham Barr, Eugene R. Bleecker, Richard C. Boucher, Russell P. Bowler, Elizabeth E. Carretta, Stephanie A. Christenson, Alejandro P. Comellas, Christopher B. Cooper, David J. Couper, Gerard J. Criner, Ronald G. Crystal, Jeffrey L. Curtis, Claire M. Doerschuk, Mark T. Dransfield, Christine M. Freeman, Mei Lan K. Han, Nadia N. Hansel, Annette T. Hastie, Eric A. Hoffman, Robert J. Kaner, Richard E. Kanner, Eric C. Kleerup, Jerry A. Krishnan, Lisa M. LaVange, Stephen C. Lazarus, Fernando J. Martinez, Deborah A. Meyers, John D. Newell, Elizabeth C. Oelsner, Wanda K. O’Neal, Robert Paine, Nirupama Putcha, Stephen I. Rennard, Donald P. Tashkin, Mary Beth Scholand, J. Michael Wells, Robert A. Wise, Prescott G. Woodruff

**Affiliations:** 1 Division of Pulmonary, Allergy, and Critical Care Medicine, University of Alabama at Birmingham, Birmingham, AL USA; 2 UAB Lung Health Center, Birmingham, AL USA; 3 Birmingham VA Medical Center, Birmingham, AL USA; 4 Division of Pulmonary Diseases and Critical Care Medicine, University of North Carolina at Chapel Hill, Chapel Hill, NC USA; 5 Department of Medicine, University of North Carolina at Chapel HillMarsico Lung Institute/Cystic Fibrosis Research Center, Chapel Hill, NC USA; 6 Gillings School of Public Health, University of North Carolina at Chapel Hill, Chapel Hill, NC USA; 7 Department of Radiology, University of Iowa Carver College of Medicine, Iowa City, IA USA; 8 Division of Pulmonary and Critical Care Medicine, University of California-Los Angeles David Geffen School of Medicine, Los Angeles, CA USA; 9 Division of Pulmonary and Critical Care Medicine, University of Michigan-Ann Arbor, Ann Arbor, MI USA; 10 VA Ann Arbor Healthcare System, Ann Arbor, MI USA; 11 Division of Pulmonary and Critical Care Medicine, Johns Hopkins University, Baltimore, MD USA; 12 Bayer AG, AG, Pharmaceutical Division, Wuppertal, Germany

**Keywords:** COPD, Acetyl proline-glycine-proline (AcPGP), Sputum, Matrikine, Inflammation, Biomarker

## Abstract

**Background:**

Pulmonary and systemic inflammation are central features of chronic obstructive pulmonary disease (COPD). Previous studies have demonstrated relationships between biologically active extracellular matrix components, or matrikines, and COPD pathogenesis. We studied the relationships between the matrikine acetyl-proline-glycine-proline (AcPGP) in sputum and plasma and clinical features of COPD.

**Methods:**

Sputum and plasma samples were obtained from COPD participants in the SPIROMICS cohort at enrollment. AcPGP was isolated using solid phase extraction and measured by mass spectrometry. Demographics, spirometry, quality of life questionnaires, and quantitative computed tomography (CT) imaging with parametric response mapping (PRM) were obtained at baseline. Severe COPD exacerbations were recorded at 1-year of prospective follow-up. We used linear and logistic regression models to measure associations between AcPGP and features of COPD, and Kaplan-Meier analyses to measure time-to-first severe exacerbation.

**Results:**

The 182 COPD participants in the analysis were 66 ± 8 years old, 62% male, 84% White race, and 39% were current smokers. AcPGP concentrations were 0.61 ± 1.89 ng/mL (mean ± SD) in sputum and 0.60 ± 1.13 ng/mL in plasma. In adjusted linear regression models, sputum AcPGP was associated with FEV_1_/FVC, spirometric GOLD stage, PRM-small airways disease, and PRM-emphysema. Sputum AcPGP also correlated with severe AECOPD, and elevated sputum AcPGP was associated with shorter time-to-first severe COPD exacerbation. In contrast, plasma AcPGP was not associated with symptoms, pulmonary function, or severe exacerbation risk.

**Conclusions:**

In COPD, sputum but not plasma AcPGP concentrations are associated with the severity of airflow limitation, small airways disease, emphysema, and risk for severe AECOPD at 1-year of follow-up.

**Trial registration:**

ClinicalTrials.gov: NCT01969344 (SPIROMICS).

## Introduction

Chronic obstructive pulmonary disease (COPD) is a progressive inflammatory disease affecting the airways and lung parenchyma, often as the result of chronic cigarette smoking. COPD is classified based on symptoms, lung function impairment, and risk for acute exacerbations of COPD (AECOPD) [1]. There remains a need to identify biomarkers that reflect the underlying molecular and cellular processes responsible for development of lung destruction (i.e. emphysema and airway remodeling) and clinical features including rapid lung function decline, chronic bronchitis, and exacerbation risk [2].

We have previously identified a matrikine (a biologically active extracellular matrix peptide), acetylated proline-glycine-proline (AcPGP), as a pathogenic regulator of cigarette-smoke-mediated emphysema development in animal models and human smokers [3, 4]. In this pathway, AcPGP is generated by the stepwise proteolytic cleavage of collagen by matrix metalloproteases (MMPs) and prolyl endopeptidase (PE) and avoids degradation by direct effects of cigarette-smoke on leukotriene A4 hydrolase [3, 5–7]. AcPGP, as a matrikine, stimulates neutrophil recruitment to sites of inflammation in the lung and propagates a feed-forward cycle of inflammation. Although our previous work has clearly demonstrated the role of the AcPGP-pathway in disease pathogenesis [3, 4], evaluation of the relevance of AcPGP in longitudinal examination of clinical features of COPD including pulmonary function, CT features, symptoms, and exacerbations is required. Additionally, our previous work has primarily focused on measuring AcPGP in the sputum [4, 8], while little focus has been put on evaluating the relevance of plasma AcPGP in COPD.

We hypothesized that the matrikine AcPGP, as a marker of lung inflammation, will be associated with meaningful clinical features of COPD including lung function impairment, emphysema, symptoms, and risk for AECOPD. We tested this hypothesis using plasma and sputum samples from participants with COPD enrolled in the SubPopulations and InteRmediate Outcome Measures in COPD Study (SPIROMICS) cohort.

## Methods

### Subjects

The design of SPIROMICS (ClinicalTrials.gov NCT01969344) has been described [9]. Briefly, SPIROMICS is a multi-center prospective observational study to identify unique biomarkers and phenotypes that can be used as intermediate outcomes to reliably predict clinical benefits in future clinical trials. SPIROMICS enrolled participants between November 2011 and January 2015. COPD was defined as a post-bronchodilator forced expiratory volume in 1-s (FEV_1_) / forced vital capacity (FVC) < 0.70 [10]. Participants underwent baseline and in-person follow-up visit 12-months later. Clinical data reported here include results from the SPIROMICS Core5 dataset. For these studies, we report data from subjects with COPD, complete clinical information, and blood and sputum AcPGP measurements. This study was approved by the University of Alabama at Birmingham IRB (X110921005).

### Blood and sputum collection and processing

Participants with a post-bronchodilator FEV_1_ ≥ 35% predicted were eligible to undergo sputum induction using nebulized saline solutions administered via ultrasonic nebulizer as previously described [11] and as outlined in the Supplementary Methods. Briefly, the saline solutions were given in three 7-min intervals and sputum was immediately processed using a 1:4 (weight:volume) 0.1% sputolysin solution followed by an additional 1:4 (volume:volume) 1 mM EDTA solution. Plasma was collected in tubes containing EDTA and was immediately processed and shipped to the Genomics and Informatics Center (GIC) at the University of North Carolina at Chapel Hill (UNC).

### AcPGP measurement

Plasma was prepared by solid phase extraction using Phree Phospholipid Removal Columns (Phenomenex, Torrence, CA, USA). First, columns were washed with a methanol:acetonitrile (60:40) solution. Next, an internal standard peptide (IS) (^13C,15N^PGP/^13C,15N^AcPGP) mixture was added to the plasma sample; plasma and IS were then placed on the Phree column. Columns were centrifuged at 4 °C for 60 min at 1300 xG followed by an additional methanol:acetonitrile wash, re-centrifugation, and collection. Afterwards, samples underwent evaporation using a Nitrogen evaporator. Dried plasma samples were then reconstituted using PBS. Sputum samples were prepared as follows: 10,000 kDa molecular weight cutoff filters were prepared by washing with an ethanol:water (65:45) solution. Next, IS was added to the sputum sample; this sputum-IS mixture was then added to the washed filters and centrifuged at 4 °C for 30 min at 12,500 xG. Finally, samples were washed using 1 mM HCl followed by centrifugation and collection of supernatants. AcPGP was measured by tandem mass spectrometry (MS/MS) as previously described [3, 4, 7, 8, 12].

### Pulmonary function

Pulmonary function testing was performed according to the SPIROMICS protocol and ATS/ERS criteria [9, 10] and post-bronchodilator values were recorded using a KoKo spirometer (nSpire Health, Longmont, Co.). Participants were stratified according to Global Initiative for Chronic Obstructive Lung Disease (GOLD) stage [1].

### Health status and respiratory symptoms

We assessed health status and quality of life using the COPD Assessment Test (CAT) [13] and the St. George’s Respiratory Questionnaire (SGRQ) [14], and dyspnea using the Modified Medical Research Council Questionnaire (MMRC) [15]. Chronic bronchitis was defined by answers to chronic cough and phlegm questions on the SGRQ [16]. Six minute walk tests were performed following ATS guidelines [17].

### Severe exacerbations

Given the impact of severe AECOPD on re-hospitalization and mortality [18], we evaluated associations between these events and sputum AcPGP. Prior severe AECOPD was defined as self-reported hospitalization for AECOPD that occurred within the 12-months preceding the baseline study visit. Prospective severe AECOPD were recorded from the time of the baseline study visit through the first year of follow-up in SPIROMICS. Severe AECOPD were self-reported during quarterly phone calls and were defined as a worsening of respiratory symptoms lasting longer than 48 h that warranted an emergency department visit or hospitalization for treatment of acute respiratory disease [19]. During the phone calls, participants (or their representatives) were asked “Since your last [visit or phone contact on [date], have you had a flare-up of chest trouble?”; positive responses were followed up with questions ascertaining the number of events, treatments for each episode (antibiotics, steroids, both, unsure, or can’t remember); participants were asked “Were you evaluated in an Emergency Department?” followed by questions on treatments; participants were then asked “Were you admitted to the hospital?” and further information was collected about dates and location of the medical facility as well as treatments given during the hospitalization.

### Radiologic measurements

The methods for quantitative computed tomography (CT) were published previously [20]. Briefly, inspiratory and expiratory lung CT scans were performed at the baseline SPIROMICS visit. Parametric Response Mapping (PRM) (Imbio, Minneapolis, MN) was used to calculate amounts of emphysema (PRM^emph^) and functional small airways disease (PRM^fSAD^) as previously defined [21, 22]. This technique was recently demonstrated to correlate significantly with histologically-confirmed small airways disease in lung specimens from patients with advanced COPD [23].

### Statistical analyses

Due to the study design, there were only 2 participants with GOLD spirometry stage 4 who underwent sputum induction; therefore, spirometric GOLD stages 3 and 4 were combined into a single group defined as severe airflow obstruction. Sputum and plasma AcPGP was divided into quartiles; elevated sputum or plasma AcPGP were defined as values above the median. Descriptive statistics, including means and standard deviations for continuous data, frequencies and percentages for categorical data, were calculated for all study variables of interest. Bivariate analyses were conducted by using the unpaired t-test for normally distributed continuous variables, Wilcoxon rank-sum test for continuous variables that were not normally distributed, or the chi-square test for categorical variables. Spearman’s rho was used to measure correlations between sputum AcPGP and lung function measured by pulmonary function testing. Analysis of variance (ANOVA) was used to compare sputum AcPGP values across spirometric GOLD stages. Associations between AcPGP and pulmonary function tests were explored using linear regression models adjusted for age, sex, and current smoking status. Additional linear regression models were further adjusted for FEV1 percent predicted to measure associations between AcPGP and quality of life assessments and quantitative CT measurements. To identify associations between sputum or plasma AcPGP and any severe AECOPD at 1-year of follow-up, we used logistic regression models, adjusted for age, sex, FEV_1_ percent predicted, prior severe AECOPD (within one year before enrollment), and current smoking status. Kaplan-Meier survival analysis with log-rank test was used to identify time-to-first severe AECOPD based on the presence or absence of elevated sputum AcPGP. All statistical tests were two-sided and were performed using a significance level of *P* < 0.05. Statistical analyses were conducted using SPSS software (Version 23, IBM Corporation).

## Results

### Characteristics of the participants

We measured sputum and plasma AcPGP at the baseline SPIROMICS visit in 271 participants, including 182 subjects with COPD. The CONSORT diagram is shown in Additional file 1 Figure S1. We limited this analysis to participants with COPD; information on the excluded participants without COPD is shown in Additional file 1. Participants with COPD were 66 ± 8 years old (mean ± SD), 62% male, 84% white race, had a post-bronchodilator FEV_1_ percent predicted 68 ± 21, and 39% were current smokers (Table 1**)**. Among COPD subjects, concentrations of AcPGP were 0.60 ± 1.13 ng/mL in plasma and 0.61 ± 1.89 ng/mL in sputum. Subjects were generally symptomatic, with CAT scores of 14 ± 8, SGRQ scores of 33 ± 19, and more than 50% had chronic bronchitis. Ten percent (*n* = 18) had a self-reported previous severe AECOPD in the previous 12-months before the baseline visit.

**Table 1:** Baseline Characteristics

	Cohort (*n* = 182)
Age, years	66 ± 8
Male sex	112 (62%)
White race	153 (84%)
FEV1, percent predicted	68 ± 21
FVC, percent predicted	94 ± 19
FEV1/FVC	0.54 ± 0.11
GOLD Stage	
GOLD 1 GOLD 2 GOLD 3 GOLD 4	48 (26.4%) 88 (48.4%) 44 (24.2%) 2 (1.0%)
Current Smoker	70 (39%)
Pack-year history	51 ± 21
CAT	14 ± 8
SGRQ score, total	33 ± 19
CB-SGRQ	101/173 (56%)
MMRC dyspnea score	1 [0–1]
6-min walk distance, m	412 ± 101
PRM-emph (%)	7.3 ± 10.4
PRM-fSAD (%)	23.4 ± 11.7
Severe AECOPD in the previous year	18/175 (10%)
Plasma AcPGP ng/ml	0.60 ± 1.13
Sputum AcPGP ng/ml	0.61 ± 1.89

Data expressed as mean ± S.D.; median [IQR]; or n (%)

### Sputum AcPGP and COPD symptoms and severity

**Fig. 1:**
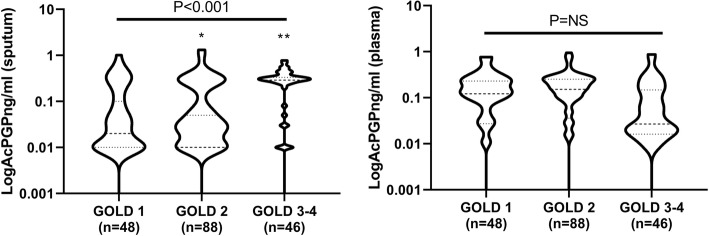
Associations between AcPGP and COPD severity. Log-transformed AcPGP in A) sputum was significantly higher in GOLD 3/4 COPD compared to GOLD 1 or 2 but there were no differences in plasma AcPGP across spirometric GOLD stages. 1-way ANOVA with Tukey’s post-hoc testing was used for analyses; *P = 0.0019 between GOLD 1 and GOLD 3/4; **P < 0.0001 between GOLD 2 and GOLD 3/4

Participants with severe airflow obstruction (GOLD spirometry stage 3–4) had higher mean sputum AcPGP concentrations compared to individuals with mild-to-moderate obstruction (GOLD 1–2; 0.98 ± 1.02 vs 0.52 ± 2.11 ng/ml; *P* = 0.05). There were no associations between sputum AcPGP and GOLD 2017 (ABCD) stages. There were significant correlations between sputum AcPGP quartiles and spirometric GOLD stage (Spearman’s Rho = 0.20; *P* = 0.007), FEV_1_ percent predicted (Spearman’s rho = − 0.14, *P* = 0.041), and FEF25–75% predicted (Spearman’s rho = − 0.18, *P* = 0.016), but not with FVC percent predicted (*P* = 0.49) or FEV1/FVC (*P* = 0.069). The distribution of log-transformed AcPGP in sputum and plasma across spirometric GOLD stages is shown in Fig. 1. Sputum AcPGP was not correlated to mean sputum neutrophil count (*P* = 0.14).

In multiple linear regression models adjusted for age, sex, and current smoking status, sputum AcPGP remained independently associated with FEV_1_/FVC, FEF25–75, and spirometric GOLD stage (Table 2). Sputum AcPGP was associated with PRM^emph^ (Beta 1.72, SE 0.70, *P* = 0.015) and PRM^fSAD^ (Beta 1.67, SE 0.81, *P* = 0.040) in similarly adjusted linear regression models. In separate models adjusting for the above covariates plus FEV1%, associations between sputum AcPGP and PRM-emphysema remained statistically significant.

**Table 2:** Associations between Sputum AcPGP and clinical features of COPD

	Model 1			Model 2^#^		
	Beta	S.E.	*P*-value	Beta	S.E.	P-value
FEV1, percent predicted	−2.58	1.38	0.063	n/a	n/a	n/a
FEV1/FVC	−0.02	0.007	0.018	n/a	n/a	n/a
FEF25–75, percent predicted	−2.40	1.20	0.046	n/a	n/a	n/a
GOLD Stage	0.14	0.05	0.004	n/a	n/a	n/a
CAT	0.93	0.52	0.073	0.55	0.49	0.27
PRM-emph	1.72	0.70	0.015	1.26	0.63	0.040
PRM-fSAD	1.67	0.81	0.040	1.08	0.70	0.127

Model 1 = Linear regression models included sputum AcPGP, age, sex, current smoking status. ^#^Model 2 = Linear regression model was adjusted for FEV_1_ percent predicted in addition to previously listed covariates (model 2 was not used for spirometric based outcome variables)

Sputum AcPGP was not associated with health status as measured by CAT, SGRQ (data not shown), or with dyspnea as measured by MMRC scores (data not shown). Nor was there a difference in mean sputum AcPGP among individuals with chronic bronchitis compared to those without chronic bronchitic symptoms (0.53 ± 1.18 versus 0.79 ± 2.78 ng/mL, *P* = 0.44 by Chi square testing).

### Sputum AcPGP and severe exacerbations

**Fig. 2:**
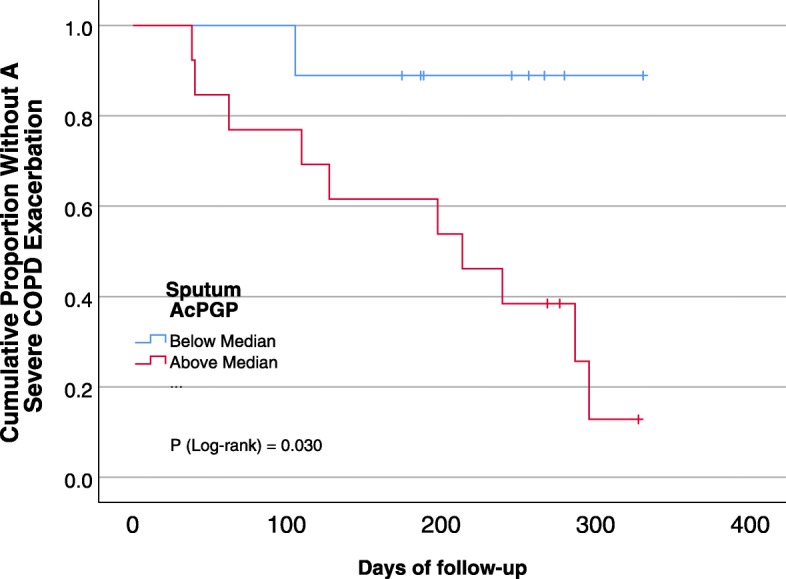
Kaplan-Meier curve for severe AECOPD. COPD subjects with elevated sputum AcPGP had shorter time-to-first severe AECOPD compared to individuals with non-elevated sputum AcPGP (195 days [95%CI 139–252] versus 305 days [259–351], *P* = 0.030)

After 1-year of follow-up, 10% (*n* = 18/173) individuals had at least one severe AECOPD (median 0 events/year, range 0–3). Although sputum AcPGP was not statistically significantly different in participants that had a self-reported prior severe AECOPD (*P* = 0.21), sputum AcPGP was significantly associated with a severe AECOPD at 1-year of follow-up (*P* = 0.019) (Table 3). Likewise, 83% (15/18) of participants that had a severe AECOPD during follow-up had sputum AcPGP above the median values compared to 45% (69/155) who did not have a severe AECOPD (*P* = 0.002). In multivariable logistic regression models adjusted for age, FEV_1_% predicted, prior severe AECOPD, and current smoking status (Table 4), for each higher quartile sputum AcPGP value there were 75% increased odds (OR 1.75; 95% CI 1.04–2.97, *P* = 0.037) of having a severe AECOPD in the next year; alternatively, if the baseline sputum AcPGP was above the median, there was a 486% increased odds of a subsequent severe AECOPD (OR 4.86; 95% CI 1.28–18.4, *P* = 0.02). Participants with elevated sputum AcPGP (above the median) also had shorter time-to-first severe AECOPD compared to individuals with non-elevated sputum AcPGP (195 days [95% CI 139–252] versus 305 days [259–351], *P* = 0.030 by log-rank test) (Fig. 2).

**Table 3:** Associations between AcPGP and COPD exacerbations

	No severe AECOPD (*n* = 155)	Severe AECOPD (n = 18)	*P*-value
Sputum AcPGP			0.019
Q1 Q2 Q3 Q4	42 (28%) 41 (27%) 38 (25%) 31 (20%)	2 (11%) 1 (6%) 7 (39%) 8 (44%)	
Sputum AcPGP above median	69 (45%)	15 (83%)	0.002
Plasma AcPGP			0.96
Q1 Q2 Q3 Q4	37 (24%) 38 (25%) 41 (27%) 39 (25%)	5 (28%) 4 (22%) 4 (22%) 5 (28%)	
Plasma AcPGP above median	73 (47%)	11 (61%)	0.27

Data expressed as n (percent). Chi square testing was used for analyses

**Table 4:** Associations between AcPGP and Severe COPD exacerbations

	Unadjusted			Adjusted		
	OR	95% CI	*P*-value	OR	95% CI	*P*-value
Sputum AcPGP (quartiles)	2.03	1.21–3.40	0.007	1.75	1.04–2.97	0.037
Elevated sputum AcPGP (above median)	6.01	1.67–21.6	0.006	4.86	1.28–18.4	0.020
Plasma AcPGP (quartiles)	0.98	0.63–1.51	0.92	1.01	0.65–1.58	0.95
Elevated plasma AcPGP (above median)	0.94	0.35–2.49	0.90	1.18	0.41–3.3	0.77

Logistic regression models were adjusted for age, sex, FEV1 percent predicted, prior severe AECOPD, and current smoking status

### Plasma AcPGP and clinical outcomes

Sputum and plasma AcPGP concentrations in individual participants were not significantly correlated (R = -0.07; *P* = 0.82). Unlike the associations observed between sputum AcPGP and features of COPD, plasma AcPGP was not associated with pulmonary function, GOLD stage, symptoms or quality of life, or risk for severe AECOPD (Fig. 1b; Tables 2-4).

## Discussion

Results of this analysis of a sizeable prospective cohort provide the first direct evidence that matrikines present in the sputum of patients with COPD are associated with salient, measurable parameters that impact disease progression and morbidity. We clearly demonstrate that induced sputum AcPGP was related to more severe airflow limitation, emphysema, and small airways disease. Additionally, we observed that sputum AcPGP was associated with a significantly increased risk for severe AECOPD within the first year of follow-up when adjusted for known risk factors including previous severe exacerbation. Sputum AcPGP was not associated with respiratory symptoms or respiratory health status measurements. We did not observe any relationships between circulating AcPGP and COPD outcomes.

These findings provide clinically relevant evidence that AcPGP has the potential to serve as a biomarker for COPD, extending previous studies showing that AcPGP is present in sputum and bronchoalveolar lavage samples in individuals with COPD as compared to healthy controls or smokers without COPD [4, 8]. Identification of measurable endpoints including pulmonary function, findings on CT imaging, and COPD exacerbations are vital to understanding the translational significance of biological pathways implicated in COPD pathogenesis. These observations provide links between mechanisms of neutrophil chemotaxis, pulmonary inflammation, and alveolar destruction and relevant translational endpoints. Our findings support sputum AcPGP as an indicator of COPD severity as well as a marker of increased odds for AECOPD development. Importantly, we have shown that sputum AcPGP is responsive to the use of daily azithromycin and roflumilast, two oral anti-inflammatory agents used for AECOPD risk reduction [8, 12]. In both studies, treatment with either agent reduced sputum AcPGP as compared to individuals treated with placebo. Although in the MACRO study we previously observed temporal rise and fall in sputum AcPGP related to the time of an AECOPD, we did not find any correlation between AcPGP and pulmonary function or exacerbation risk, possibly due to the small sample size [12]. The current study addresses this gap in understanding the clinical relevance of this matrikine by directly linking it to lung structure/remodeling, pulmonary function, and prospective exacerbation risk.

The utility of sputum versus blood-based biomarkers in COPD is the subject of debate and ongoing investigation [2]. On one hand, blood-based assays are attractive to clinicians and investigators due to the wide availability of samples, ease in collection, standardized methodology, costs, and quality control issues as compared to induced sputum acquisition. However, it is not clear that blood-based biomarkers accurately reflect the active pathologic processes occurring in the lungs. One of the first major examples of this phenomenon in COPD was described by Singh and colleagues in the Evaluation of COPD Longitudinally to Identify Predictive Surrogate Endpoints (ECLIPSE) study. In that analysis, the investigators measured associations between sputum neutrophils as a potential biomarker for COPD among 488 participants [24]. They found sputum neutrophils were associated with pulmonary function and health status, but no association between sputum neutrophils and AECOPD or emphysema, suggesting a role for sputum based biomarkers in COPD. Likewise, Hastie and colleagues reported high degree of discordance between blood and sputum eosinophils in SPIROMICS, with sputum eosinophilic inflammation being a more robust biomarker for disease severity, exacerbation frequency, and more quantitative CT emphysema than blood eosinophils [25]. As in our current study, neither group found robust associations between sputum and blood biomarkers. Although systemic markers are commonly consider to result from excess inflammation in the lung milieu that spillover into the circulation, it is increasingly recognized that markers present in pulmonary and systemic compartments may result from separate mechanisms [26]. Hence, our unexpected lack of relationships between plasma AcPGP and COPD outcomes in the current study may indicate that circulating AcPGP reflect processes unrelated to COPD. In addition to the neutrophil-chemoattractant properties of AcPGP, there is increasing evidence that AcPGP plays critical roles in endothelial dysfunction, angiogenesis, and cardiovascular injury [27, 28]. Thus, circulating AcPGP may reflect a cardiac or pulmonary vascular disease, conditions that are highly prevalent in COPD and should be studied in the future. Nevertheless, this work provides additional support for continuing pursuit of sputum-based biomarker panels for COPD.

Our study has limitations that deserve mention. First, participants with very severe COPD were excluded from sputum induction given concerns of safety in advanced disease. Thus, our findings reflect a moderate-to-severe COPD population, which remains highly relevant given that this group accounts for most individuals with COPD. Another limitation is the lack of a validation cohort. While this diminishes the generalizability of the findings, we and others have demonstrated the biological relevance of this matrikine pathway in the pathogenesis of COPD. Additionally, we only analyzed sputum AcPGP at one time point. However, we have previously shown that sputum AcPGP values remain consistent when measured repeatedly over a 12-week period [8]. Further, the small number of severe exacerbations increases the risk for type 1 error in our observations of increased odds of severe AECOPD and elevated sputum AcPGP. These findings should be validated in other cohorts. Finally, because of the observational nature of the study, we cannot determine the causality of the associations between elevated sputum AcPGP and features of COPD. Nevertheless, the known properties of AcPGP suggest that it warrants investigation as a potential therapeutic target to modify COPD progression.

## Conclusions

Our data support sputum but not systemic AcPGP as associated with the severity of airflow limitation, emphysema and small airways disease, and risk for severe exacerbations in established COPD. Future prospective studies are needed to better elucidate the impact of elevated pulmonary AcPGP in patients at high risk for disease progression or exacerbations.

## Supplementary information

**Additional file 1.** Supplementary Methods. **Figure S1.** CONSORT Diagram.

## Data Availability

Interested investigators may request access to available data through processes outlined on the SPIROMICS website (https://www.spiromics.org/spiromics/) under Obtaining SPIROMICS Data.

## Abbreviations

AcPGP: Acetylated proline-glycine-proline
AECOPD: Acute exacerbation of COPD
CAT: COPD Assessment test
CB-SGRQ: Chronic Bronchitis defined by the SGRQ
FEV1: Forced expiratory volume in 1-s
FVC: Forced vital capacity
GOLD: Global initiative for chronic obstructive lung disease
MMRC: Modified medical research council
PRM-emph: Percent emphysema measured by parametric response mapping
PRM-fSAD: The percent functional small airways disease measured by parametric response mapping
SGRQ: St. George’s Respiratory Questionnaire
