# Supplementary material for: The matrikine acetyl-proline-glycine-proline and clinical features of COPD: findings from SPIROMICS
Source: Respir Res. 2019 Nov 12;20:254. doi: 10.1186/s12931-019-1230-8 (PMC6852714; doi:10.1186/s12931-019-1230-8)

**Additional file 1: Supplementary Methods**

***Sputum Induction and Processing:***

All staff performing sputum induction and processing were trained and certified by the Genomics and Informatics Center (GIC) at the University of North Carolina at Chapel Hill. SPIROMICS participants whose post-bronchodilator FEV1% predicted was ≥35% were eligible to participate in sputum induction. Sputum was induced in three 7-minute intervals using an ultrasonic nebulizer. Spirometry was performed 2-minutes after the start of saline nebulization at each step. If the subject’s FEV1 dropped more than 20% of predicted at any step or the subject complained of breathing discomfort, the induction process was stopped. If the FEV1 did not drop >10%, the saline concentration was increased for the subsequent session. Subjects with a post-bronchodilator FEV1% predicted ≥50% underwent sputum induction using a protocol of sequential 3%, 4%, and 5% saline solutions. Individuals with a post-bronchodilator FEV1% predicted ≥35% but <50% underwent a protocol using 0.9%, 3%, and 3% saline solutions.

Sputum samples were expectorated after each 7 min interval, or at the end of a procedure terminated before completion. Sputum samples were immediately processed. After removing aliquots for mucin, microbiome, and viscoelasticity measurements, samples were weighed and diluted with a 1:4 (weight:volume) ratio of 10% Sputolysin solution and were rocked for 15 minutes at room temperature. The sample was further diluted with an equal 4-fold volume of 1mM EDTA and rocked for an additional 5 minutes. The sample was next filtered through a 48-52 μm nylon mesh filter. The filtrate was centrifuged at 500xg for 10 minutes to pellet cells. Supernatant fluid was dispensed into aliquots for cytokine examination. The cell pellet was resuspended in HBSS and had cell count and Cytospin slide preparation performed. Cytospin slides were stained, dried, coverslips applied and slides shipped to the central sputum slide reading center (University of NC, Chapel Hill, NC). Any remaining cells were pelleted, and resuspended in 1 ml of Trizol reagent with 10 μl of GGD. Supernatant aliquots and cell pellet were stored at -80°C until shipped to the central biospecimen repository at University of North Carolina, Chapel Hill, NC.

**Supplementary Results**

*Description of the non-COPD sample*

The 89 SPIROMICS participants without COPD were 59±10 years old, 53% male, 77% white race, had a post-bronchodilator FEV1 percent predicted 97±11%, and 38% were current smokers. Sputum AcPGP was 0.16±0.66 ng/ml and plasma AcPGP was 0.74±1.87 ng/ml for the non-COPD cohort. Sputum AcPGP was lower in the non-COPD controls compared to the COPD group (P=0.030) but plasma AcPGP did not differ based on the presence or absence of COPD (P=0.47).

**Figure S1. CONSORT Diagram**


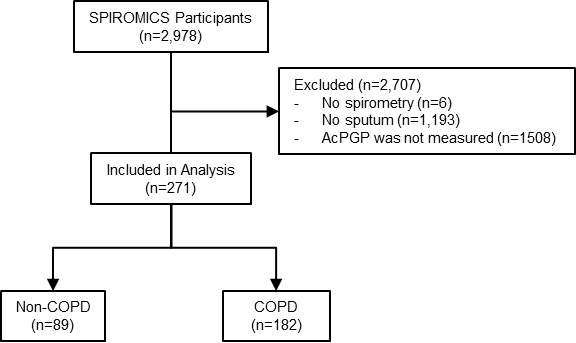

Supplement: Supplementary file 1 — Additional file 1. Supplementary Methods. Figure S1. CONSORT Diagram. [file 12931_2019_1230_MOESM1_ESM.docx]
